# On the Material Constitutive Behavior of the Aortic Root in Patients with Transcatheter Aortic Valve Implantation

**DOI:** 10.1007/s13239-023-00699-7

**Published:** 2023-11-20

**Authors:** Chiara Catalano, Tahir Turgut, Omar Zahalka, Nils Götzen, Stefano Cannata, Giovanni Gentile, Valentina Agnese, Caterina Gandolfo, Salvatore Pasta

**Affiliations:** 1 Department of Engineering, Università degli Studi di Palermo, Viale delle Scienze, Palermo, Italy; 2 4RealSim Services BV, Groene Dijk 2B, 3401 NJ IJsselstein, The Netherlands; 3 Department for the Treatment and Study of Cardiothoracic Diseases and Cardiothoracic Transplantation, IRCCS-ISMETT, Palermo, Italy; 4 Radiology Unit, Department of Diagnostic and Therapeutic Services, IRCCS-ISMETT, Palermo, Italy; 5 3D printing and Virtual Reality Laboratory, Department of Research, IRCCS-ISMETT, IRCCS Mediterranean Institute for Transplantation and Advanced Specialized Therapies, Via Tricomi, 5, Palermo, Italy

**Keywords:** Transcatheter aortic valve replacement, Finite element analysis, Inverse analysis, Transcatheter heart valve

## Abstract

**Background:**

Transcatheter aortic valve implantation (TAVI) is a minimally invasive procedure used to treat patients with severe aortic valve stenosis. However, there is limited knowledge on the material properties of the aortic root in TAVI patients, and this can impact the credibility of computer simulations. This study aimed to develop a non-invasive inverse approach for estimating reliable material constituents for the aortic root and calcified valve leaflets in patients undergoing TAVI.

**Methods:**

The identification of material parameters is based on the simultaneous minimization of two cost functions, which define the difference between model predictions and cardiac-gated CT measurements of the aortic wall and valve orifice area. Validation of the inverse analysis output was performed comparing the numerical predictions with actual CT shapes and post-TAVI measures of implanted device diameter.

**Results:**

A good agreement of the peak systolic shape of the aortic wall was found between simulations and imaging, with similarity index in the range in the range of 83.7% to 91.5% for n.20 patients. Not any statistical difference was observed between predictions and CT measures of orifice area for the stenotic aortic valve. After TAVI simulations, the measurements of SAPIEN 3 Ultra (S3) device diameter were in agreement with those from post-TAVI angio-CT imaging. A sensitivity analysis demonstrated a modest impact on the S3 diameters when altering the elastic material property of the aortic wall in the range of inverse analysis solution.

**Conclusions:**

Overall, this study demonstrates the feasibility and potential benefits of using non-invasive imaging techniques and computational modeling to estimate material properties in patients undergoing TAVI.

## Introduction

Transcatheter aortic valve implantation (TAVI) has become an accepted alternative to open-chest surgery [1], and computational models such as finite element analysis (FEA) and fluid-solid interaction have been developed in the last decades to simulate this procedure [2–5]. These simulation techniques allow for virtual deployment of the transcatheter heart valve in patient-specific models and provide insights into the device-host interaction. Structural parameters can be estimated from numerical simulations to quantify the performance of the implanted device [3] whereas hemodynamic quantification enables an estimation of critical TAVI-related parameters such as the paravalvular leakage [6]. In other words, computational modeling could serve to support safe planning of structural valve interventions and present new insights in the post-procedural care.

However, simulations should be carried out at an increasingly challenging level to address the need for verifying and validating the model output [7]. A reliable and accurate computational model of TAVI should account for patient-specific material properties. Despite the promising findings of current computational techniques [8–10], the actual material properties of the aortic root in TAVI patients remain unknown and uncertain. This lack of knowledge is mainly caused by the fact that TAVI is routinely carried out as a non-invasive technique in elderly patients at high surgical risk [11]. This means that tissue segments cannot be collected for ex-vivo biomechanical material evaluation, and current material descriptors are not reliable due to age-related changes that likely occur on the vessel wall. As a result, there are no realistic material properties available for TAVI patients that can be used for numerical simulations. Inverse analysis, when contrasted with direct finite-element simulations, emerges as a valuable avenue for estimating material parameters within intricate cardiovascular contexts. This methodology finds applicability in diverse scenarios, including the estimation of material properties for the human aorta under both healthy [12] and pathological states [13, 14]. However, it is noteworthy that the numerical validation of inverse analysis, particularly concerning comparisons with in-vitro or animal models, has not been thoroughly addressed. This facet warrants due consideration as it plays a pivotal role in establishing the reliability and robustness of the inverse analysis technique in practical scenarios beyond computational simulations.

This study aims to estimate patient-specific material properties of the aortic root and calcified valve leaflets non-invasively using an inverse analysis approach and cardiac gating computed tomography angiography (angio-CT). The current lack of reliable material properties for TAVI patients is addressed by optimizing a linear-elastic material constitutive relationship in patient-specific models using regression analysis, which minimizes the difference between predictions and CT-based measurements of the aortic wall strain and valve orifice area. The proposed material calibration strategy is validated at two levels. Before TAVI, the numerical predictions of the vessel systolic shape and valve orifice area were compared to the actual angio-CT counterpart. After TAVI simulations, predicted device diameters were compared to CT-based measurements. A sensitivity analysis assessing the impact of the elastic material descriptor on the TAVI simulation output was also performed.

## Methods

### Patient Population and Image Analysis

Twenty patients, with age ranging from 75 to 91 years, whom underwent TAVI with the 23-mm SAPIEN 3 Ultra (S3) device (Edwards Lifescience, Irvine, USA) were enrolled in this study (see Table 1). All patients were enrolled in the study after obtaining ethical approval and informed consent from the IRCCS ISMETT hospital. Demographic data and brachial cuff pressure measurements were collected at in-hospital admission whereas the function of the stenotic aortic valve was evaluated by Doppler echocardiography. Angio-CT imaging with contrast-agent was carried out to compute the aortic valve annulus and thus for the pre-planning of the optimal device size. CT imaging had spatial resolution of resolution 0.488 × 0.488 × 0.625 mm. TAVI was performed by transfemoral access under general anesthesia, with the S3 device facing the left ventricle for one-third of its device length. No pre-dilation or device overexpansion was carried out. For the purpose of this study, a post-TAVI angio-CT imaging was carried out one month after the structural valve intervention.

**Table 1:** Clinical demographic, CT-related measurement and Jaccard index and Young modulus after optimization

	Age (years)	Sex	P_sys_ (mmHg)	P_dias_ (mmHg)	HR (bpm)	CO (ml/min)	Strain_Ann_ (%)	Strain_Sinus_ (%)	Strain_STJ_ (%)	Orifice area (mm^2^)	JI (%)	E (MPa)
#1	79	M	162.0	48.0	92.0	5336.0	3.4	1.4	4.8	140.2	91.5	6.1
#2	90	F	140.0	75.0	87.0	4611.0	3.6	0.7	5.3	86.5	86.9	6.2
#3	85	F	160.0	90.0	65.0	3174.0	1.8	3.1	0.3	119.0	90.1	6.2
#4	83	F	143.0	60.0	67.0	4690.0	5.8	5.2	1.9	87.8	83.7	6.3
#5	86	F	107.0	59.0	91.0	5005.0	7.4	8.6	3.7	105.4	85.2	5.2
#6	85	F	150.0	52.0	61.0	3416.0	8.0	3.6	6.8	70.8	86.9	3.8
#7	85	F	138.0	78.0	74.0	2442.0	2.0	1.8	1.8	119.8	88.6	6.2
#8	89	M	120.0	58.0	63.0	3591.0	1.7	1.8	4.3	112.8	86.7	6.2
#9	91	F	138.0	55.0	69.0	2415.0	4.6	8.5	6.1	108.4	84.2	7.7
#10	83	F	110.0	52.0	65.0	4485.0	1.9	0.4	1.8	102.2	85.3	7.4
#11	75	M	100.0	55.0	64.0	4575.0	4.3	1.6	7.2	104.3	86.7	6.2
#12	85	F	151.0	54.0	67.0	2881.0	9.1	3.6	4.0	97.8	85.9	3.4
#13	86	F	119.0	58.0	62.0	3162.0	3.5	3.7	4.2	109.4	86.5	5.9
#14	84	M	123.0	78.0	68.0	3808.0	3.2	4.1	4.4	125.7	90.3	5.0
#15	79	M	126.0	60.0	61.0	3104.0	8.2	8.5	7.9	165.2	84.9	3.9
#16	75	M	128.0	47.0	62.0	3968.0	1.8	2.1	1.8	140.8	85.7	7.5
#17	82	M	113.0	64.0	81.0	6399.0	7.4	7.7	8.2	100.8	86.9	4.9
#18	78	M	136.0	67.0	60.0	5100.0	3.5	2.8	3.1	138.6	89.9	5.9
#19	82	M	105.0	55.0	65.0	3185.0	6.2	6.1	6.4	145.2	88.5	4.9
#20	85	F	118.0	58.0	88.0	4210.0	4.8	5.1	5.2	98.5	85.1	3.6
Mean	83		129.4	61.2	70.6	3977.9	4.6	4.0	4.5	114.0	87.0	5.6
SD	4		18.3	11.2	10.9	1046.3	2.4	2.7	2.2	23.1	2.2	1.3

*M/F* male/female, *P*_*sys*_ systolic pressure, *P*_*dias*_ diastolic pressure, *HR*  heart rate, *CO* cardiac output, *JI* Jaccard Index

The Mimics medical imaging software (v21, Materialise, Belgium) was adopted to reconstruct the aortic wall including the aortic root, valve leaflets and calcifications as done in similar studies [15, 16]. Specifically, angio-CT images at end-diastole were used for the segmentation process. The aortic wall was initially segmented by semi-automatic thresholding, and then manual editing and smoothing were used to finalize the anatomy. The segmentation of bright calcifications was carried out using grey intensity values, with a fully-automatic approach consisting of specific intensity thresholds to distinguish between calcified and non-calcified regions. Stenotic valve leaflets were modeled using anatomic measurements and 3rd-order NURBS curves in Rhinoceros software (Rhinoceros v.7, McNeel & associates, USA) [17]. In brief, leaflet free edges were manually segmented by spline curves in the axial plane after multiplanar reformations of diastolic images. The leaflet-to-sinus attachments were identified by spline curves generated on the aortic root surface. Each leaflet belly was modeled using a curve that was constrained on both the leaflet-free-edge and leaflet-to-sinus curves. A single control point at mid-level was employed for modeling the curvature of native valve leaflets using a spline curve. The leaflet-to-sinus curves were projected onto the aortic root surface, and then the final shape of the native valve leaflets was developed using a multi-patch surface network.

### Patient-Specific and S3 Model Development

To enhance the credibility of TAVI simulations, the patient-specific model was constructed in accordance with ASME V&V40 standards. A separate study is dedicated to describing the numerical verification of the proposed computational framework [18]. In brief, each anatomic part was meshed using a grid refinement that ensured a relative error of < 1.0% on the output parameter of interest (i.e., maximum principal stress). The discretization error analysis also evaluated the element type (triangular versus quadrilateral) and element formulation (reduced or full integration) on the resulting patient-specific model output. The aortic wall was meshed using triangular shell elements (S3R) with a size of 0.8 mm, while the calcification was meshed using tetrahedron solid elements (C3D4) with a size of 0.5 mm. For native valve leaflets, an unstructured prismatic mesh (C3D6) was developed by extruding triangular shell elements (size of 0.6 mm) with four layers through-the-thickness. Uniform thickness was assumed for the patient model, as it cannot be measured from CT imaging for either the aortic wall or the valve leaflets. Specifically, the aortic root was assumed to be 2 mm thick, while the native valve leaflets had a uniform thickness of 0.5 mm [10, 19]. Discretization analysis was performed for four patients, and the lowest mesh refinement value for each anatomic component was determined to be the optimal set for the entire patient population.

A linear-elastic material model was assumed for the biomechanical response of the aortic wall and aortic valve leaflets, with Poisson’s coefficient of ν = 0.475 and Young’s moduli estimated by the inverse analysis. A neo-Hookean material model was assumed for the calcific plaques using C_10_ = 67.7 MPa and D1 = 7.5E−3 MPa [20].

As for the patient-specific model, the development of S3 model was verified according to ASME V&V40 standards. Specifically, the device model was developed through an analysis of various modeling techniques and element mesh types to optimize the computational time while preserving the device structural performance. The stent frame was modeled with surface elements to describe the outer device skin as well as beam elements to represent the device skeleton (see Appendix 1 for additional details). The simplification in device modeling is justified by focusing on the main point of interest: the radial stiffness of the stent frame, rather than the high-resolution stress-strain state in the strut cross-section. Nevertheless, it's important to note that this simplification might yield results different from those obtained using a model with solid elements. While the overall radial stiffness of the device model using solid elements differs from that of the current approach (specifically, 79.89 N/mm for the present model versus 93.51 N/mm for solid elements), there is only a marginal variance in device diameter after simulating the recoil and expansion of the bioprosthesis (<1%). After discretization analysis, the S3 geometry was meshed with 22,974 surface elements (SFM3D4R) tied to 5718 beam elements (B31). The cobalt-chromium material behavior of the stent frame was described using a combination of isotropic elasticity and Johnson-Cook plasticity to account for hardening and rate material dependence. The elastic model had a Young's modulus of 238.54 GPa and Poisson's coefficient of 0.29. The plasticity model was described by yield stress of A = 465.0 MPa, hardening parameter of B = 2140 MPa and coefficient of n = 0.73, and density of 7650 kg/m^3^ The valve leaflets related to the S3 model were obtained through a forming simulation process. This involved constructing the leaflets in a planar position and then manipulating them into their final functional shape using a sequence of geometric operations, as described by Bailey et al. [21]. Thus, the device valve leaflets were meshed with 1457 structured elements (C3D8R) and one solid-element layer through-the-thickness with full integration. The Ogden constitutive law with 2^nd^ order polynomial form was assumed to model the biomechanical response of the pericardial tissue using μ_1_ = 0.96 MPa, α1 = − 56.5, μ_2_ = 3.57 MPa, α2 = 1.87, and D1 = 0.027 MPa [22]. The device skirt was modeled with a neo-Hookean material model (i.e., C_10_ = 1.7 MPa and D1 = 0.65 MPa) [23] and was tied to the stent frame to mimic the device suture. To mitigate undesired high-frequency oscillations, a Rayleigh damping factor of 250 was applied to the device skirt, and a viscous pressure of 6.55E−06 MPa was used on the inner surface of the valve leaflets. The balloon was discretized with 82,322 unstructured shell elements (S3), with geometry extrapolated from Bailey et al.[21] and neo-Hookean material properties (C_10_ = 36.5 MPa and D1 = 1.36E−3 MPa).

### Inverse Approach

**Fig. 1:**
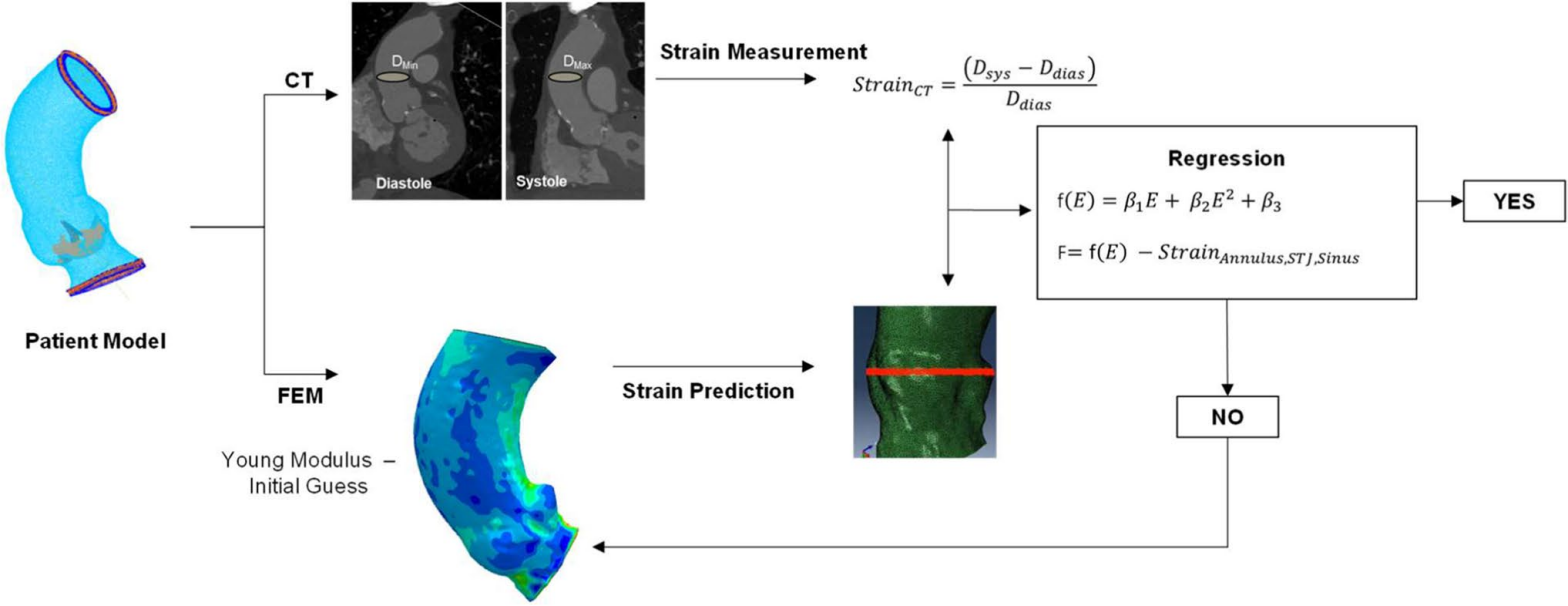
Flowchart of the inverse analysis approach for material property estimation

The inverse analysis aims to obtain the material descriptors that minimize the difference of both the vessel strain and aortic valve orifice area between FEAs and actual imaging measurements (see Fig. 1). The inverse approach assumes a regression model to link the input variables (i.e., the material properties) to the output variables (i.e., the aortic wall strain or the orifice area for the stenotic valve). Specifically, the variable of interest for the aortic wall was the peak systolic strain as measured by ${(D}_{sys-}{D}_{dias})/{D}_{dias}$ where ${D}_{sys}$ and ${D}_{dias}$ are the systolic and diastolic aortic diameters measured from the angio-CT scan. For the aortic valve, the orifice area was computed at peak systole in the aortic valve plane. Given the assumption of linear elastic material properties, the unknown material parameters can be represented solely by the elastic moduli of both the aortic wall and native valve leaflets. The minimization was performed using a least-squares method assuming the following quadratic regression model:

1 $$f\left(E\right)={\beta }_{1}+{\beta }_{2}E+{\beta }_{3}{E}^{2},$$

where $E$ is the unknown constitutive parameter and $\beta$ are the regression coefficients. For each patient, 15 FEAs were performed to simulate the cardiac cycle with the material properties randomly varied within the range of 0.8–15 MPa for each simulation. The range was selected to encompass a diverse set of material parameters, spanning from stiff to compliant material properties as suggested by Bosi et al. [20]. Thus, the nominal strain at peak systole was exported at different anatomic levels, which were identified by several element sets at the annulus, sinus, and sino-tubular junction (STJ). To calculate the valve orifice area, a Python script was developed in the Rhinoceros CAD software. The script automatically extracted the nodal coordinates of the valve leaflet free edges and then calculated the valve orifice area by spline interpolation of the extracted points. Simulations were carried out in the Abaqus\Explicit solver (v.2021hf7, Dassault Systèmes, FR). The distal ends of the aortic wall were constrained in the longitudinal direction using cylindrical coordinate systems, whereas tie contact constraints were used to constrain the calcification to the native valve leaflets. Cuff pressure measurements and echocardiographic evaluations of pressure gradients across the stenotic valve were employed as boundary conditions for the aortic wall and valve leaflets, respectively. Specifically, a physiological pressure waveform was imposed on the aortic wall by proportionally scaling systolic/diastolic pressures and beat timing based on cuff pressure measurements and individual heart rates of each patient. For the valve leaflets, the peak gradient pressure obtained from echocardiography was directly applied to the patient model's valve leaflets.

Following FEAs, a matrix linking the input (i.e., material parameters for each random value of the Young’s modulus) and the output (i.e., systolic strain/orifice area) was implemented in Matlab software (v.2021a, MathWorks Inc). Therefore, the cost function for both the aortic wall and calcified valve leaflets can be defined as:

2 $$\begin{aligned} F&=f\left(E\right)-\text{Strain}_{\rm annulus, sinus, STJ} \\ F&=f\left(E\right) - \text{Orifice\; Area}\end{aligned}$$

These cost functions should converge to zero, indicating that the output variable predicted by the regression function for each patient matches the corresponding value measured in angio-CT imaging at end-diastole. If the optimization yields two different sets of material parameters, the one falling within the predefined range of elastic modulus variation is selected as the optimal set. If both values are within the range, only one value is chosen as the optimal material parameter for the aortic root. Specifically, we retained the material parameter leading to positive value (i.e., stretched) of the aortic wall strain or valve orifice area.

To verify the results of the inverse analysis, a new simulation of the cardiac beat was conducted using the optimal material parameters for both the aortic wall and valve leaflets. The deformed shape of the aortic wall at peak systole was then compared to its actual CT counterpart using the Jaccard index to quantify the level of agreement among vessel shapes. For native valve leaflets, the orifice area predicted with the optimal material parameters was compared to the imaging-based measurements.

### TAVI Simulation

**Fig. 2:**
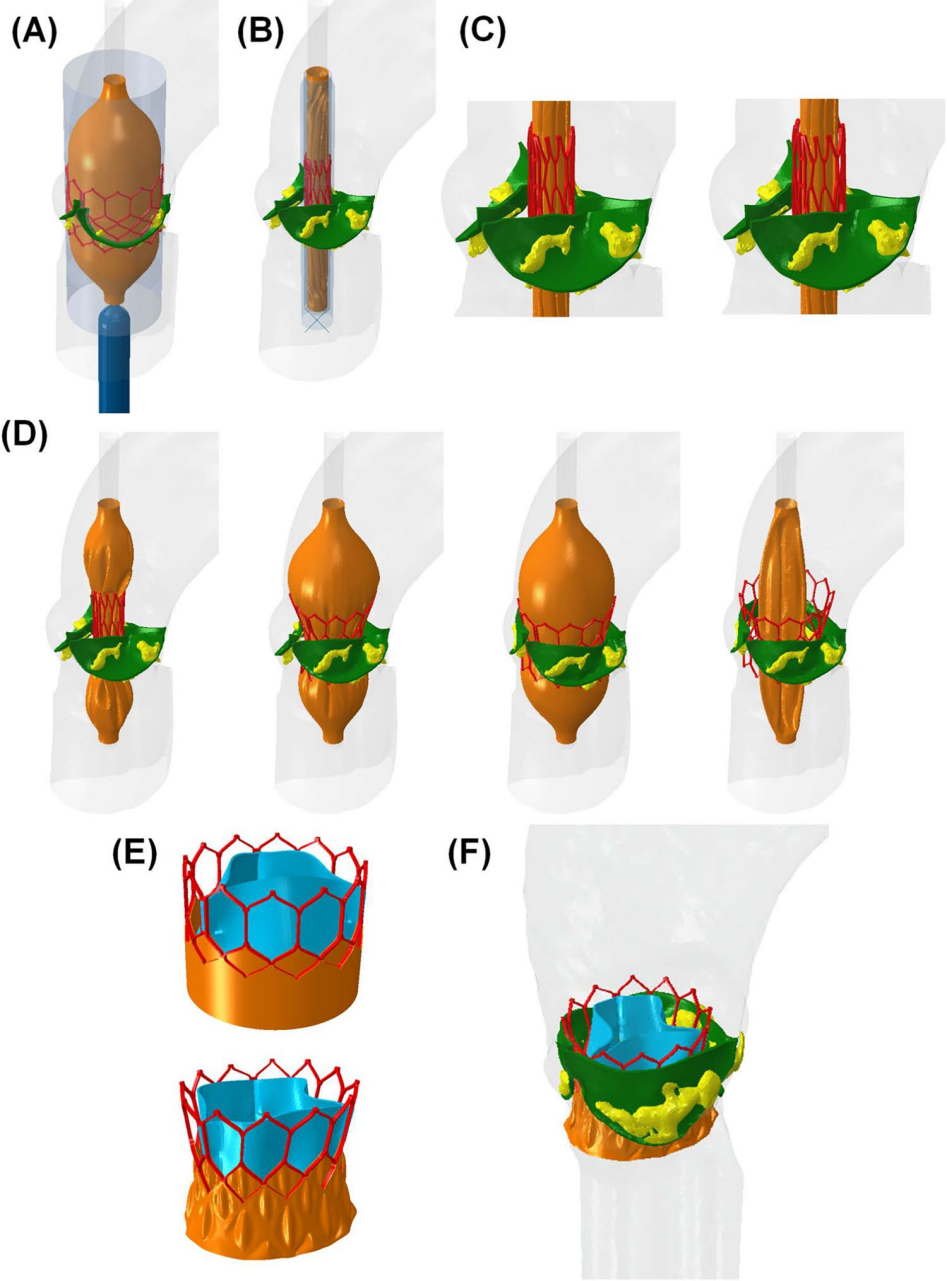
Step of TAVI simulation including **A** undeformed configuration, **B** crimping of the device and opening of stenotic leaflets, **C** S3 stent frame before and after elastic recoil, **D** implantation from balloon inflation ending to deflation, **E** device skirt and valve leaflets mapping on deformed stent-frame, and **F** final TAVI simulation model

The TAVI-related FEA was performed using the Abaqus/Explicit solver and a quasi-static approach to model dynamic phenomena. The S3 delivery system was placed within the aortic root anatomy, with the distal end of the balloon radiopaque marker aligned with the circumference of the aortic annulus *via* three hinge points on the annulus. The balloon axis was positioned perpendicular to the aortic valve plane, and the implantation depth was determined from post-TAVI angio-CT imaging to place the S3 system along the vessel centerline. The TAVI simulation involved simulating the device crimping and recoil, followed by deployment of the S3 in the calcified valve leaflets, as previously done [9]. During the first step (see Fig. 2A), the S3 stent frame was crimped by means of a cylindrical surface using displacement boundary conditions to reduce the device diameter to the nominal value of 6.7 mm. Simultaneously, the calcific valve leaflets were pushed away by a punching surface to avoid element overclosures (time step of 0.5 s). Then, the elastic recoil was allowed by removing the contact definition between the crimping surface and the stent frame for a time period of 0.1 s. During the implantation step (i.e., 0.5 s), the balloon was inflated upon the nominal fluid volume of 17 ml using the fluid cavity approach in combination with a VUAMP subroutine that relates the stent expansion to the nominal diameter with the inflation volume. Contact conditions with penalty factors were activated between the patient model and S3 system for mimicking the TAVI procedure in the human host. For the S3, the contact condition was generated for the surface skin rather than the beam skeleton. To prevent any undesired penetration when assembling the device skirt and leaflets, a fourth step was adopted to push away the calcification and native valve leaflets using a cylindrical surface and zero-velocity boundary conditions for the stent frame to retain the implanted device shape. This was possible since linear-elastic material properties were adopted for each anatomic part of the patient-specific model. Thus, the device skirt and valve leaflets can be imported and linked to the stent frame by first imposing nodal displacement from the first simulation step, and then generating tie contact conditions between the stent frame and its components. The surface used to displace the native valve leaflets and calcification is displaced back into the original place, causing the S3 to come into contact with the patient-specific model.

The FEA included the analysis of the numerical solver error for determining the computational setting according to ASME V&V40 standards. This analysis aimed to evaluate the impact of varying numerical solver parameters on the model response output. Specifically, the investigated solver parameters were: (i) Rayleigh damping, (ii) viscous pressure, (iii) bulk viscosity, (iv) mass scaling, (v) contact parameters, and (vi) penalty factors. The influence of these parameters was evaluated based on the relative error in the device output response, and the best set of parameters was identified for relative errors < 1%. We also performed a numerical code verification to ensure the reliability of the proposed computational model in solving the discrete equations governing the physics of the TAVI problem. To achieve this, a representative benchmark problem was identified for each component of the patient-specific model and S3 system and then compared to an analytical solution. For instance, the LaPlace law was used as the analytical solution of the aortic root wall, which was compared to the benchmark problem of a cylindrical shell under uniform pressure. The benchmark problem had the same element type, mesh refinement, boundary conditions, and other solver parameters adopted for the patient-specific model. Details of these analyses, which were conducted to estabilish the credibility of the proposed computational model, are described by Catalano et al. [18].

To further validate the results of inverse analysis, the predicted device configurations were compared to that seen at post-TAVI angio-CT imaging. This involved measuring the S3 diameter at the inflow, mid, and outflow cross-section levels for both simulations and CT scans. Additionally, a sensitivity analysis on the aortic wall material parameter was conducted for four patients by imposing the minimum and maximum values of the elastic modulus resulting from the solutions of the regression model at different aortic regions (i.e., annulus, sinus, and STJ). The effect of these changes on the resulting S3 device diameter was quantified by the relative error with respect to the undeformed configuration.

## Results

**Fig. 3:**
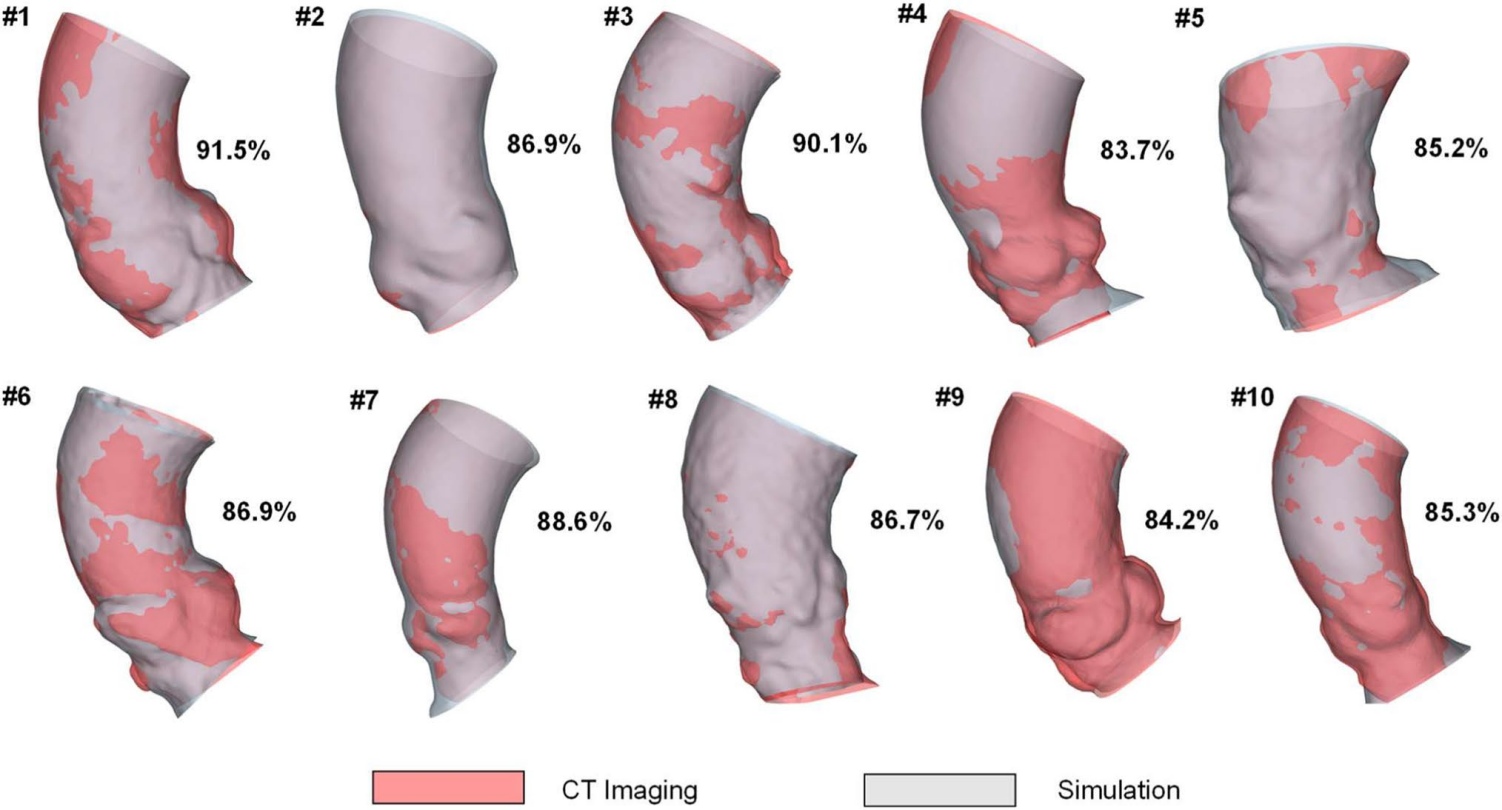
Comparison of peak systolic shape of the aortic root between predictions and angio-CT imaging; values of Jaccard index are reported

The deformed shapes of the aortic wall, obtained from simulations of the cardiac beat with optimal Young’s modulus, were compared to those from pre-TAVI angio-CT segmentations at peak systole (see Fig. 3). The highest difference was qualitatively observed in the region of the left ventricular outflow tract, likely due to the heart kinematics stretching and twisting the diseased aortic root. Nonetheless, the Jaccard index revealed a good level of agreement between predictions and image-based shapes, indicating a similarity in the range of 83.7% to 91.5% across the patient study group.

**Fig. 4:**
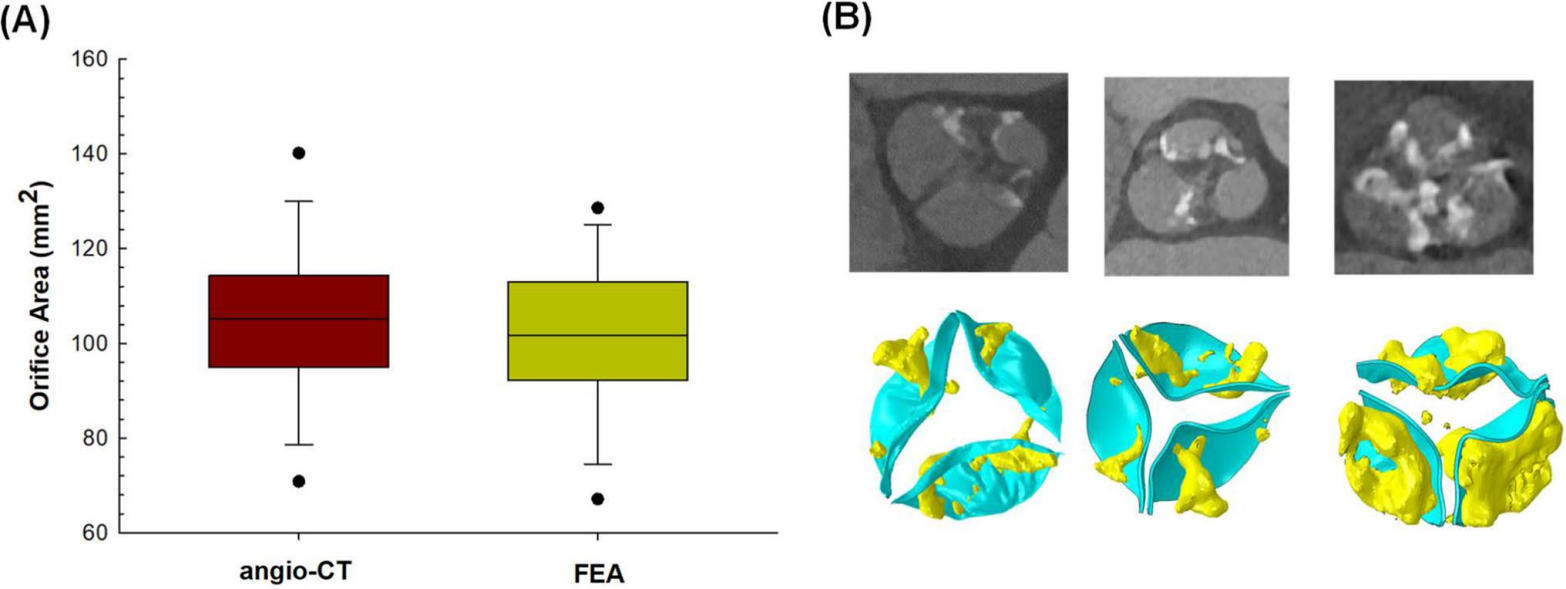
**A** Box plot for comparison of predicted- versus CT-based measurements of the orifice area at peak systole and **B** qualitative comparison between simulation and CT images of calcified valve

A box plot graph was adopted to compare predictions of valve orifice area with the optimal material model as compared to those of pre-TAVI angio-CT imaging (see Fig. 4A). No statistical difference was found between the predictions and imaging measurements. Figure 4B displays the opened configuration of the calcified valve at peak systole for three representative patients with moderate to severe calcification volumes.

**Fig. 5:**
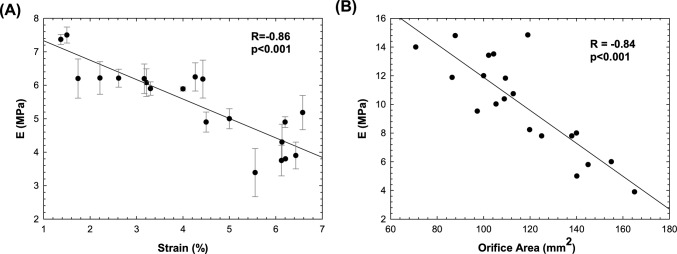
Correlation analysis for **A** the aortic wall between the optimal elastic modulus and CT-based strain measurement of the vessel and **B** for the stenotic valve between the optimal elastic modulus and CT-based measurements of the orifice area; error bars indicates the standard error mean of estimated elastic modulus for the aortic wall

At Pearson’s analysis, a negative linear relationship of the optimal elastic parameters for each patient with the strain measured from the pre-TAVI angio-CT was observed (R = − 0.86 and p < 0.001, Fig. 5A). Similarly, the measurement of orifice area done after reformatting of CT scan in the valve plane were negatively correlated to the elastic material parameter used for the native valve leaflets (R = − 0.84 and p < 0.001).

**Fig. 6:**
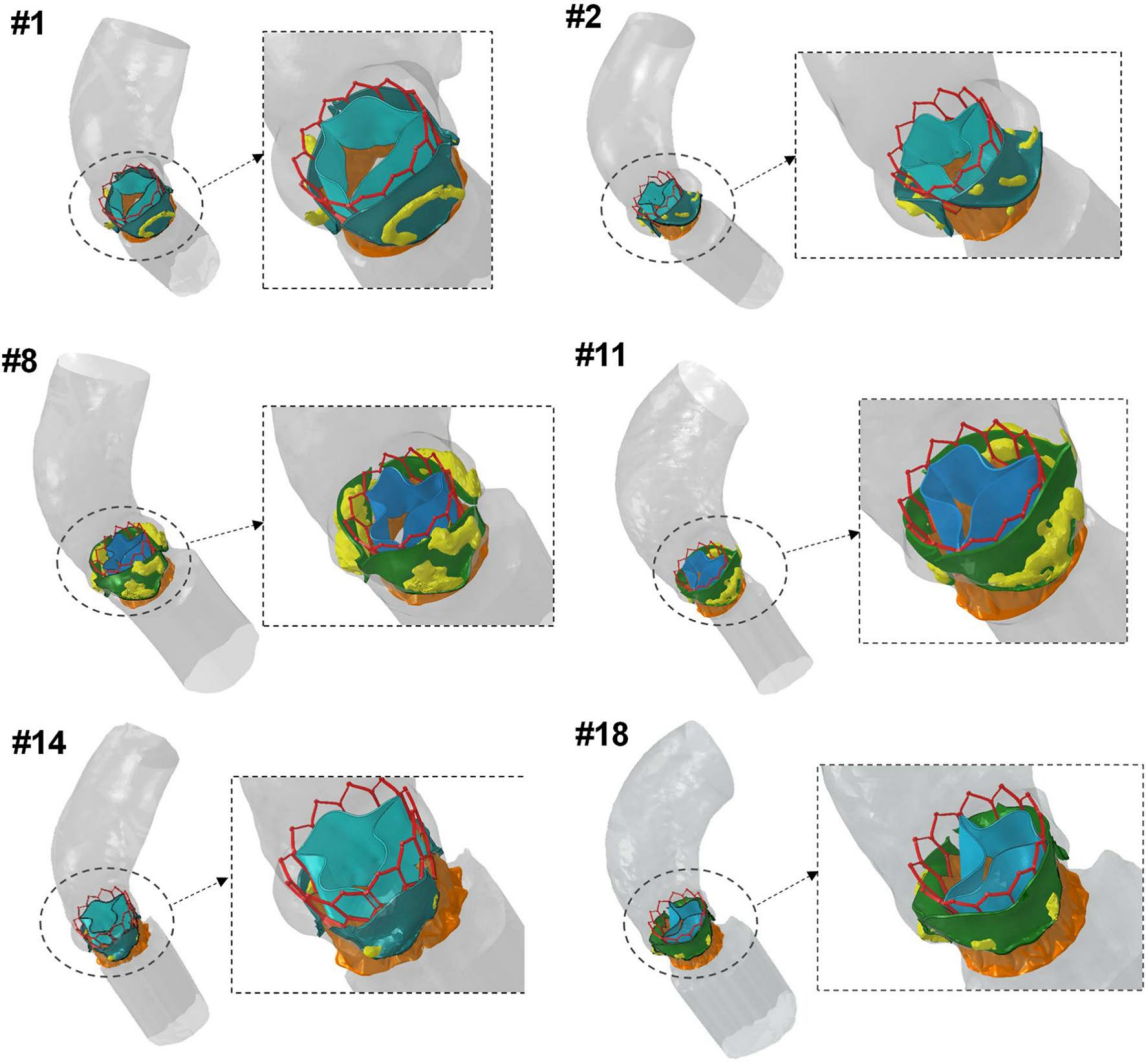
Representative final configuration resulting from TAVI simulation in different patients: patients #1, #14 and #18 had mild stenosis, patient #2 had bicuspid aortic valve; patients #8 and #11 had severe stenosis

**Fig. 7:**
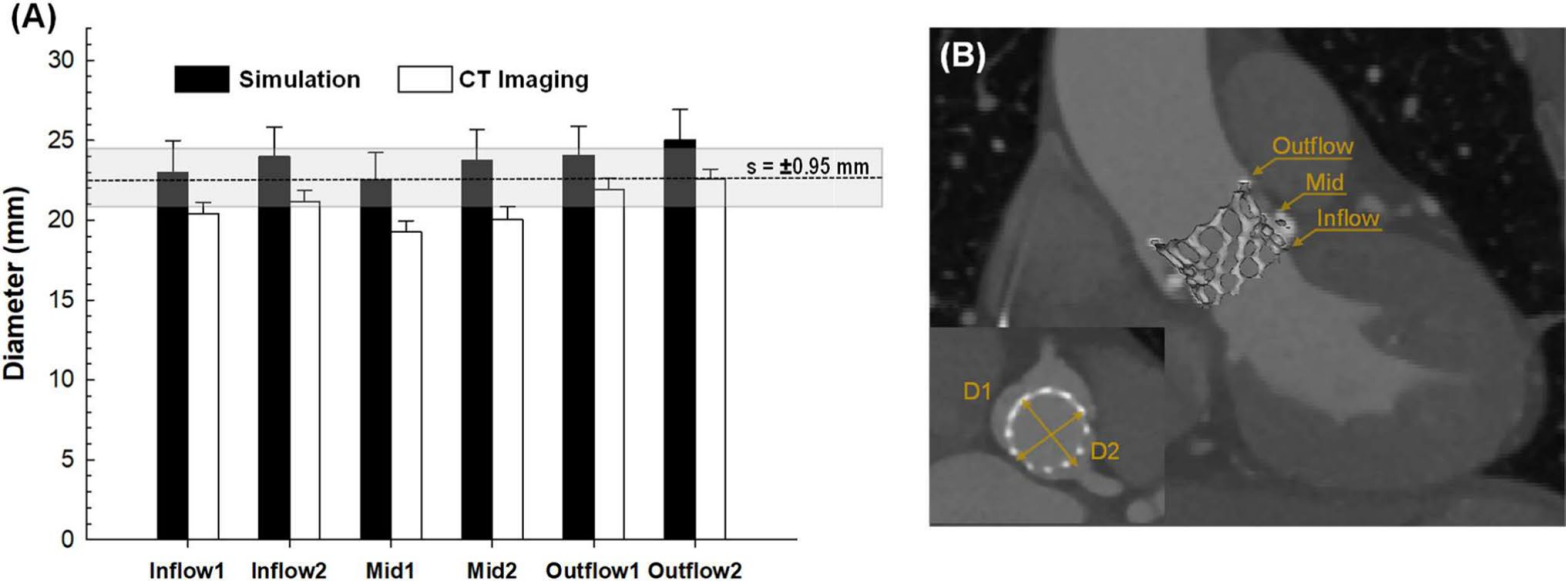
**A** Bar comparison of S3 device diameter at different anatomic levels between computational simulations (black bar) and CT measures (white bar); subscripts 1 and 2 indicates maximum and minimum outer device diameters; **B** post-TAVI angio-CT image for a representative patient showing locations of diameter measurement

**Fig. 8:**
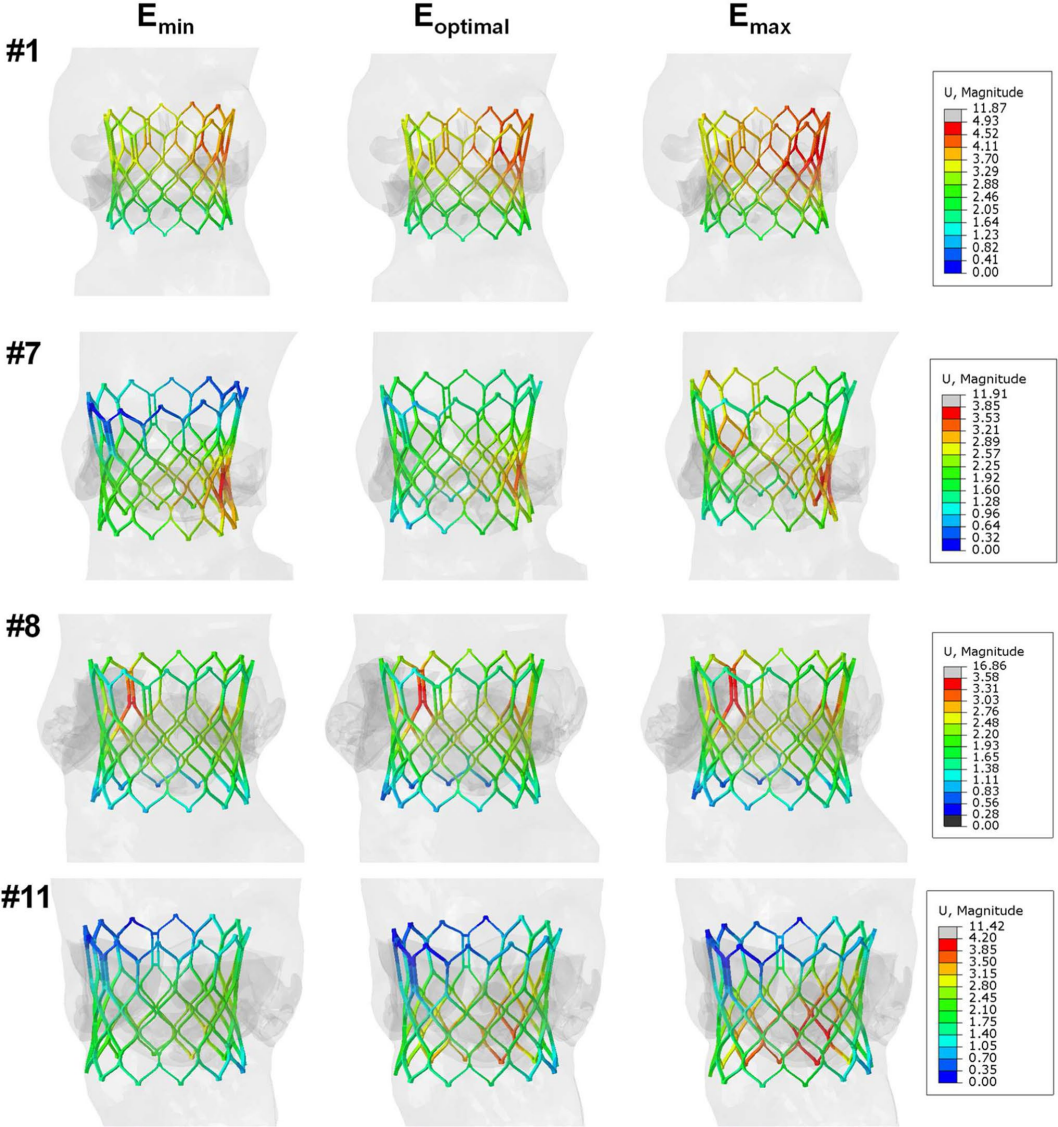
Maps of the displacement field of S3 device with respect to the undeformed shape for minimum, optimal and maximum values resulting from the inverse analysis; patients #1 and #11 had mild stenosis, patient #7 and #8 had severe stenosis

The deployment of the S3 device with valve leaflets in the free-stress configuration varied among patients with different anatomies and degrees of calcification, as shown in Fig. 6. To validate the proposed material model, we conducted a comparison between the minimum and maximum external measurements of the predicted S3 diameters and those acquired from CT imaging at different anatomical levels (refer to Fig. 7A). The distribution of predicted S3 diameters did not differ statistically from the imaging-based measurements, with relative errors in diameter changes ranging from 6.1 to 10.2%. The grey band in the bar plot indicates the average thickness of the 23-mm S3 device to account for artifacts due to the metallic nature of the stent frame. A sensitivity analysis was conducted to determine the impact of vessel material parameters on the resulting TAVI simulation, which revealed a modest change in the displacement field of the S3 stent frame (see Fig. 8). The highest elastic parameter caused the greatest displacement field of the S3 stent frame with respect to the nominal device diameter. The relative error in diameter changes computed from simulations with the highest and lowest material parameters ranged from 0.17 to 3.78%, with patients with severe calcifications showing the highest changes (see Table 2).

**Table 2:** Range of Young modulus and device diameters with relative errors in parenthesis

	E (MPa)	Inflow (mm)	Mid (mm)	Outflow (mm)
#1	4.01–8.33	23.36 (1.11)	21.24 (1.46)	24.13 (0.17)
#2	3.14–8.88	23.32 (2.47)	20.77 (1.98)	24.27 (0.82)
#5	1.20–9.70	22.41 (3.28)	20.8 (3.32)	23.83 (1.26)
#7	2.31 − 9.36	21.84 (3.78)	20.71 (2.43)	24.32 (2.15)
#8	3.55–8.87	23.46 (1.32)	20.94 (0.33)	24.49 (0.57)
#11	1.72–9.74	23.56 (2.20)	21.49 (2.56)	24.52 (1.47)

## Discussion

This study presents a novel non-invasive inverse approach for estimating reliable material properties of the aortic root and calcified valve leaflets of patients with severe aortic valve stenosis undergoing TAVI. By adopting pre-TAVI angio-CT images at two cardiac phases, the proposed inverse analysis optimizes patient-specific elastic parameters using a regression model to minimize the difference between predictions and CT-based measurements of aortic wall strain and orifice area at peak systole. Despite the simplicity of the material relationship, predicted systolic shapes of the vessel wall showed good agreement with the actual CT counterpart. Similar results were found for the opened configuration of stenotic valve leaflets between predictions and imaging measurements. Once the optimal material parameter set was achieved, TAVI was simulated to further validate the inverse analysis output by comparing the S3 device diameter between numerical predictions and post-TAVI angio-CT images. The predicted device diameters at several anatomic levels were in good agreement with those measured by medical imaging analysis, with a difference in the diameter < 10.5%. A sensitivity analysis revealed the impact of the material parameters obtained from the inverse analysis on the S3 device diameter after TAVI simulations. This proposed inverse analysis has significant implications for improving patient care as it enables the optimization of material properties in a population with advanced ages where current *ex-vivo* material descriptors are not suitable.

In this setting, Trabelsi et al. [24] demonstrated the potential of inverse analysis based on imaging data for determining the material descriptors of the two-terms Demiray’s constitutive model. As compared to our approach, they adopted three cardiac phases (i.e., the systole, mid-cycle and diastole) to quantify the unknown material parameters using the aortic wall volume as the output parameter. The inverse analysis was applied to five patients with aortic aneurysms and indicated a maximum relative error of 0.019% between numerically-predicted and CT-based measurements of the luminal vessel volume. Since the pre-TAVI angio-CT imaging procedure here adopted allows to extrapolate ten cardiac phases in the R-R interval, the present inverse approach can be extended to complex constitutive law using different parameters as the aortic volume or the deformation at different cardiac phases. Similar approaches were also implemented for abdominal aortic aneurysms using ultrasound imaging to non-invasively quantify the regional aortic wall variations of elastic material properties in combination with finite-element analyses [25, 26].

Age-related changes to the structure and composition of the aortic wall result in alterations to its biomechanical response as the aortic wall becomes stiffer and less compliant. This can lead to reduced strain and increased stress impacting the cardiovascular function. From the vascular mechanics point of view, the aging leads to a leftward shift of the stress-strain response under biaxial loading as characterized by an increased value of the physiological elastic modulus. These changes are exacerbated in patients undergoing TAVI as their age at the time of transcatheter interventions is typically in the range of 75–80 years old while the average age for patients undergoing open-chest surgery is generally lower, around 65–70 years old [11]. However, there is a lack of information on the material properties of patients with advanced age, as ex-vivo mechanical testing is impossible for this population [19, 27]. Using biaxial testing on eight fresh-frozen human cadaver hearts with ages ranging from 80 to 98 years old, Martin et al. [28] demonstrated that the human aortic tissue behaves significantly stiffer than the corresponding porcine tissues in both circumferential and longitudinal directions. These findings raise questions about the reliability of using current *ex-vivo* data to investigate the biomechanics involved in TAVI patients, thereby stimulating the development of non-invasive inverse analysis.

Recent findings using computational modeling have led us to adopt a simple linear elastic material model to capture the biomechanical response of the calcific aortic root [10, 20]. Bosi et al. [10] demonstrated good agreement between TAVI simulations and imaging measurements in a large cohort of TAVI patients when the aorta was assumed to be a linear elastic material. Post-TAVI echocardiographic data were used to validate the implantation configurations obtained by simulations of both the SAPIEN XT and CoreValve devices in the stenotic aortic valve. The reported value of Young's modulus was 7.78 MPa, which is similar to the mean value obtained with our inverse analysis (5.6 ± 1.3 MPa). It should be however observed that echocardiography has low resolution and non-uniform voxel shape. In a different way, we adopted CT imaging to validate the inverse analysis for material parameter estimation. We carried out two levels of validation to ensure the reliability of the constitutive material parameter: i) comparing the pre-TAVI predictions of the peak systolic shapes of the vessel with the actual CT counterpart; ii) comparing the post-TAVI predictions of S3 stent frame diameters with those from angio-CT imaging. These findings overall support the use of linear-elastic modeling for the biomechanical response of TAVI patients with advanced age. It should be however noticed that more complex constitutive models have been used to model the aortic root using fiber-reinforced constitutive relationship [5] or six-term polynomial form to account for the sinus material differences [2].

There are several limitations to this study. Firstly, the non-invasive inverse analysis was limited to patients who underwent TAVI with the 23-mm S3 device. The inverse analysis was applied to patients who received the 23-mm SAPIEN 3 Ultra device, thus limiting the material response to such patient population. Future analyses are necessary to warrant the material parameters on patients with different device size or self-expanding bioprosthesis. Additionally, the inverse analysis was designed to obtain a global value of the material parameters, rather than considering regional variations in the biomechanical properties of the vessel or changes in tissue thickness along the longitudinal direction of the aorta. To circumvent numerical challenges and mitigate excessive element distortion, the crimping simulation intentionally omitted the inclusion of the skirt and valve leaflets. In this setting, Bressloff's study [29] has underscored the stresses experienced by the valve leaflets when loaded to crimping forces and highlighted the challenges in conducting comprehensive simulations of crimping to achieve clinically relevant diameters, particularly when encompassing the device's valve leaflets within the simulation framework. The assumption of uniform thickness for both the aortic root and valve could influence computational outcomes, potentially leading to underestimation or overestimation of risks associated with the TAVI procedure. However, this choice has been justified by the challenge in accurately discerning the true mechanical interaction between the host and the device, and a stiff response serves as a suitable representation for the elderly TAVI population. The extraction of the free edge of stenotic valve leaflets or the measurements of aortic wall strains might be influenced by imaging resolution or image artifacts. Furthermore, there was no synchronization between the data extracted from the computational model and the CT imaging. These observations have the potential to affect the simulation output of the proposed inverse analysis. Future studies will incorporate uncertainty analyses to quantify the impact of material thickness and other assumptions on the resultant material response of patients undergoing TAVI. Moreover, fluid-solid interaction analysis will be performed to enhance the fidelity of simulating the heartbeat and provide a more accurate depiction of the dynamic behavior of the stenotic aortic valve. A validation of the proposed framework with fluoroscopy videos of the same patient will be also carried out.

## Conclusion

The proposed non-invasive inverse approach for estimating material constituents of the aortic root and calcified valve leaflets in TAVI patients is an effective method with significant implications for improving patient care. This study demonstrates good agreement between predictions and CT-based measurements of biomechanical and morphological parameters of the vessel wall before TAVI, as well as the structural configuration of the implanted device after TAVI. The use of linear elastic modeling may be sufficient for capturing the biomechanical response of TAVI patients with advanced age, while still preserving the reliability of computational predictions for TAVI simulations.

## Figures and Tables

**Fig. 9:**
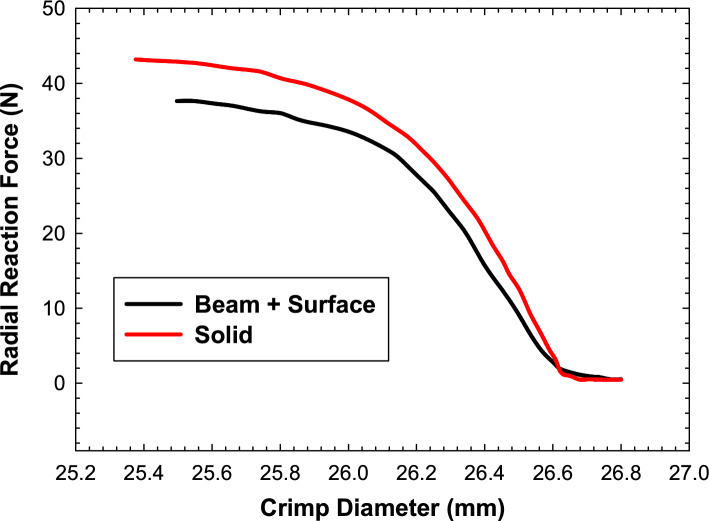
Comparison of the stent frame response between solid and surface + beam elements

## Data Availability

The datasets generated during and/or analysed during the current study are not publicly available due to ethical issues but are available from the corresponding author on reasonable request.

## Appendix 1

The stent frame of the S3 device is a thin, wire-like metallic structure with high radial stiffness, which maintains a constant opening of the diseased valve. To improve computational efficiency, this study employed a modeling approach for the S3 stent frame based on beam elements enveloped by a water-tight surface, representing the stent frame's geometry to meet the requirements of the finite volume approach. The intended use of the present model does not encompass any stress-related fatigue or damage behavior for the S3 device. Therefore, a detailed modeling of the stent frame using continuum elements was deemed unnecessary, as long as the overall stiffness and deformation behavior are accurately captured.

The use of beam elements to represent the device's structural response is justified through a detailed comparison with a continuum model. The radial stiffness of the stent frame, whether modeled with beam + surface or solid elements (S4), was evaluated through a dedicated simulation that compressed the S3 model in the radial direction, similar to crimping prior to TAVI simulation. Figure 9 illustrates the radial reaction force as a function of crimp diameter for simulations with beam + surface and solid elements. The model comparison clearly reveals an underestimation of approximately 15% in radial stiffness when the stent frame is crimped. The difference between the crimped outer diameters and expanded outer diameters is expected to be small since the stent frame is constrained by the crimp tool and balloon during crimping and expansion, respectively. At the crimp recoil state, the outer diameter is 4% lower for the beam model, and this difference is also evident in the crimp recoil percentage results (see Table 3). Importantly, at the expanded device configuration, there is only a marginal variance between crimped and expanded outer diameters. Specifically, the difference between the solid model and the proposed beam + surface element is less than 1%. This low value indicates the excellent capability of the proposed beam + surface approach to capture the overall device stiffness while maintaining computational efficiency.

**Table 3:** Comparison of S3 model response between solid and beam + solid elements

	Radial stiffness (N/mm)	R^2^	Ave.OD crimped (mm)	Ave.OD Cr. Recoil (mm)	Recoil Pct, Crimp	Ave.OD expanded (mm)	Ave.OD exp. recoil (mm)	Recoil Pct, Exp.
Solid	93.51	0.99987	4.5323	5.7896	27.74	27.1261	26.2152	3.36
Beam	79.89	0.99962	4.5448	5.5583	22.30	27.1339	26.0382	4.04
error	− 14.6%		0.3%	− 4.0%		0.0%	− 0.7%	

*Ave.OD* average outer diameter, *Recoil Pct, Crimp*  crimping recoil percentage computed as the ratio of the change in the stent outer diameter between recoil and crimping on the crimped outer diameter, *Recoil Pct, Exp.* expansion recoil percentage computed as the ratio of the change in the stent outer diameter between recoil and expansion on the expanded outer diameter
